# Risk Adjustment in Home and Community Based Services Outcome Measurement

**DOI:** 10.3389/fresc.2022.830175

**Published:** 2022-03-29

**Authors:** James Houseworth, Tina Kilaberia, Renata Ticha, Brian Abery

**Affiliations:** ^1^Institute on Community Integration, University of Minnesota Twin Cities, Minneapolis, MN, United States; ^2^Betty Irene Moore School of Nursing, University of California, Davis, Davis, CA, United States

**Keywords:** risk adjuster, disability, home and community based services, National Quality Forum, outcome measurement

## Abstract

The purpose of this study was to review and evaluate existing research that used risk adjusters in disability research. Risk adjustment controls for individual characteristics of persons when examining outcomes. We have conducted a systematic review and an evaluation of existing studies that included risk adjusters for outcomes of people with disabilities receiving services (home or community based). The process included coding each study according to the type(s) of risk adjusters employed and their relation to the specific population and outcomes within a framework. Panels were utilized to prioritize the risk adjusters. Findings indicate that four risk adjusters can be tentatively recommended as potential candidate risk adjusters: chronic conditions, functional disability, mental health status, and cognitive functioning. Holistic Health and Functioning far outweighed other outcomes studied to date. Further, there is a need for testing recommended risk adjusters across multiple outcomes and different populations of people with disabilities.

## Introduction

**Figure 1:**
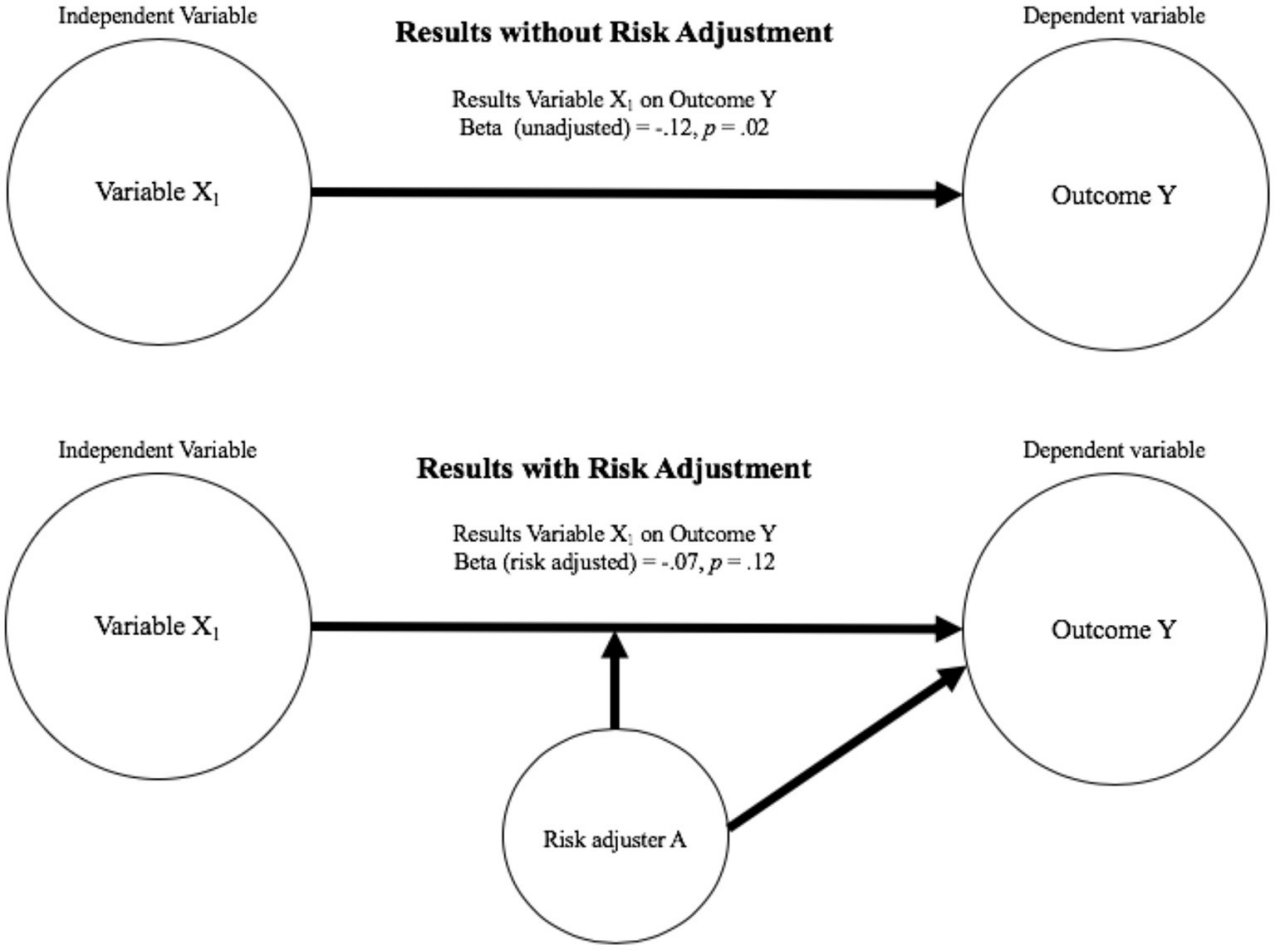
Representation of risk adjusted models.

*Risk adjustment* refers to the practice of identifying and including known or potential factors that could be significantly associated (positively or negatively) with the outcome of interest and are only indirectly related to the research/evaluation question under scrutiny (1) and are a type of covariate. Like a risk adjuster, a *covariate* is a variable associated with target outcomes but may not be of interest or only of interest to the extent to which it interacts with another variable. A risk adjuster can be thought of as a covariate that is included as a control to decrease the chances of making an error in interpreting the associations between variables one does care about. Risk adjusters are often incorporated into analyses to adjust results (e.g., magnitude estimates, etc.), and are not reported. Figure 1 represents this process. Largely, risk adjustment seeks to *level the playing field* by eliminating variance in an outcome due to individual characteristics or contextual factors outside of the control of the people being assessed or compared. When appropriately used, this adjustment can lead to enhanced equity in decisions when interpreting the results in question.

Risk adjustment is increasingly recognized as crucial to health care reimbursement and comparing provider performance in terms of the quality and outcomes of care (2). In practice, risk adjusters are not selected randomly. Risk adjustment for insurance reimbursement purposes is different than for outcome-based research. Risk adjustment for insurance reimbursement focus on creating risk scores, scores that adjust based on expectations of costs for care. Risk adjustment for outcomes research are driven by theory after consideration of factors potentially related to outcomes of interest (3). Appropriately implemented, risk adjustment allows for fair comparisons among service providers by accounting for factors beyond the purview of those delivering supports that may affect patient outcomes (e.g., person's age, and severity of disability). Within the healthcare field, risk adjustment research focuses on the complexity and difficulty of accounting for risk adjusters and making statistically-appropriate adjustments (2, 4). While this process is ubiquitous in medical reimbursement, it has the potential to improve measurement and analysis of the quality of life outcomes of recipients of home and community-based services (HCBS). Beyond these methodological benefits, the practice of risk adjustment in HCBS has the potential to lead to increased equity in both policies and decisions regarding service provision and consequently the outcomes experienced by beneficiaries.

When assessing differences in outcomes between groups of people or individuals, it is often necessary account for the characteristics of those under study (5). Research, for example, has demonstrated that age, health condition, and level of disability as well as more malleable factors (e.g., levels of service) may have an impact on the effectiveness of supports and the outcomes people experience (6).

Because a risk adjuster can influence the relationship between a predictor and an outcome, if it is not considered we may mistakenly interpret statistically significant results as indicating the presence of a relationship that in reality does not exist (Type 1 error) or miss a relationship that is of consequence (Type 2 error). The inclusion of risk adjusters not only increases the accuracy of results lowering the probability of Type 1 and Type 2 error, but is critical in terms of ethical considerations. In the absence of its use, for example regulatory agency could erroneously conclude that one support provider is underperforming when the real factors underlying perceived differences between groups are associated with one or more of the characteristics of those receiving services.

The present study considers the role of risk adjustment in the field of disability research, as related to quality of life outcomes for people with five types of disabilities (physical, intellectual and developmental, age-related and psychiatric disability as well as traumatic brain injury). In addition to summarizing current risk adjustment practices in this field, we make recommendations based on our findings as to future risk adjustment practices.

### The National Quality Forum and Risk Adjustment

The National Quality Forum (NQF), identifies risk adjustment as a critical step in evaluating outcome measures to empirically ensure threats to validity are addressed. Sociodemographic status (e.g., income, race) has been explored as a potential risk adjuster by the NQF with the goal of setting guidelines for risk adjustment. In 2017, the NQF reviewed 303 measures submitted for its endorsement to assess their suitability to risk adjust social risk factors potentially associated with specific health outcomes. The NQF panel ultimately recommended these social risk factors meet the same criteria for inclusion as clinical or health risk factors [see (7)] noting, however, the lack of a conceptual basis for including social risk adjusters.

Of particular interest to the broader topic of risk adjustment for purposes of the current study, the NQF panel recommended that sociodemographic factors included should have: (1) a conceptual relationship with the outcome of interest, (2) an empirical association with the outcome, (3) variability, (4) presence before any intervention (or care), (5) independence from any intervention or policy change, (6) resistance to change, (7) accurate data that can be feasibly obtained, (8) unique contribution to variance to the outcome, (9) potential contribution to the overall model, and (10) potential face validity and acceptability [(7), pp. 9–10]. We consider these as guidelines in differentiating risk adjusters from covariates.

More recently, the NQF (8) conducted an environmental scan and technical expert panel (TEP) on risk adjustment in an effort to develop guidance for measure developers. This work considered conceptual and statistical methods for risk adjustment identifying that the social factors used in risk adjustment were largely at the person and community level. Community factors were found to come from a variety of socioeconomic and demographic indicators. Comparatively, functional risk factors were all at the individual level and often based on survey information from the people assessed. Fewer functional risk factors were identified with a lack of consensus as to how to define functional status. In terms of statistical methods, regression analyses were most common, however other approaches (e.g., hierarchical linear modeling) were used to better incorporate a range of risk factors.

Other models of risk adjustment share common themes with the NQF panel recommendations. The Centers for Medicare and Medicaid Services (CMS) sought expert recommendations on risk adjustment. The Health and Human Services (HHS)-Operated Risk Adjustment Methodology Meeting reviewed and consolidated these recommendations into a set of ten *Principles of Risk Adjustment* (9). Five of these are similar to the NQF guidelines with recommendations that risk adjusters: (a) be clinically meaningful, (b) predict medical expenditures, (c) have adequate sample sizes, (d) encourage specific coding, and (e) “be internally consistent.”

Further support for some of the NQF guidelines comes from the Research Agency for Healthcare Research and Quality (10) who recommend that (a) risk adjustment does not include variables affected by the outcome, (b) the selection of variables be based on background knowledge about the relationship between the variable and the outcome, and (c) the risk adjusters have statistical associations with the outcome.

While we consider these guidelines when selecting risk adjusters, we caution against a “one size fits all” conception of a risk adjuster. As can be seen in the guidelines reviewed, risk adjustment is dependent on theoretical knowledge of the outcome assessed, target population and study aims. It should also be understood that the focus of the NQFs review and its expertise are in the area of *health care*. This is a decidedly different field from that of home and community-based services where outcomes are not as black and white and can rarely be validly assessed on the basis of single items or frequency counts.

### The National Quality Forum HCBS Outcome Measurement Framework

In 2006, the U.S. Department of Health and Human Services (HHS) through CMS contracted with the NQF to convene a panel to develop a framework that would guide measurement of HCBS outcomes for people with disabilities. The framework developed by the NQF committee ultimately included 11 domains (e.g., Community Inclusion, Choice and Control, etc.) and 40 subdomains. In our exploration of risk adjustment, we focus on these domains in order to explore how current risk adjustment practices can be used in evaluation and research related to these important quality of life outcomes for HCBS recipients. It should be noted, that the NQF has not engaged in any research in an attempt to validate its framework with stakeholders. However, the authors, as part of the Research and Training Center on HCBS Outcome Measurement, have conducted research with stakeholders that provides evidence of content validity of the framework (11).

### Risk Adjustment and Home and Community-Based Services Outcomes

In the field of HCBS outcome measurement, research has demonstrated that outcomes related to choice-making and job attainment are associated with both individual (12–14) and system-level factors (12, 15) suggesting that risk adjustment maybe a useful procedure to consider when examining these outcomes in this area. Even though HCBS outcomes for people with disabilities have been studied extensively, including the influence of covariates, risk adjustment has, to date, not been a common practice.

Risk adjustment in HCBS can be used to enhance informed choice when selecting providers and services and as a way to monitor system quality [see for example (2)]. The failure to take in to account important individual and systemic differences between providers (e.g., age and gender of clients served), may result in ratings providing consumers with information that is neither reliable nor valid. If such efforts at transparency and consumer choice can be combined with risk adjustment, models such as Gressel's (2) can be expanded to support more informed and equitable decision-making.

Challenges of capturing individual level variance that are result of relatively stable individual characteristics can be seen in the experience of disability itself. Conditions that are disabling differ qualitatively as well as with respect to their magnitude. In addition, some of these characteristics (e.g., cognitive capacities) can fluctuate over periods of time. In addition, disability itself entails both human factors as well as those present in the environment (16). The characteristics of person-level factors include disabilities that traverse the domains of physical and mental health, cognitive and functional conditions. Specific disabling conditions can co-occur. An individual may experience intellectual disability as well limitations associated with chronic illness. The combination of person-level factors with challenges people with disabilities experience in the environment (e.g., lack of accessibility), raises the question as to which factors to meaningfully include in risk adjustment. Monitoring disability over time adds another consideration, as disabilities may have different onset, cycle and evolution.

### Risk Adjustment and Statistical Methodology

Risk adjustment requires sophisticated statistical techniques. Generally, multivariate methods are used, including analysis of covariance (ANCOVA), regression, structural equation modeling (SEM), as risk adjustment involves evaluating the relationship between variables of interest and outcomes, while simultaneously accounting for risk factors. Used appropriately, these methods allow one to statistically account for the effect of individual factors (that play a role but are uncontrollable) permitting assessment and understanding of those effects separately from the primary factors of interest (6, 17).

### Cautions With Respect to the Use of Risk Adjustment

Although risk adjustment techniques have the capacity to improve the interpretation of results related to the outcomes experienced by HCBS beneficiaries, there are caveats. Murtaugh et al. (18) compared simple risk adjusted models for home health care quality to more complex models that employed a stepwise approach. They found similar results but noted there are advantages to simpler models that use risk adjustment to a lesser extent. These trade-offs are particularly important to consider when dealing with small datasets as the inclusion of too many risk adjusters can obscure relationships by spreading variance across multiple variables. It is also important to consider that one loses explanatory power when increasing the number of risk adjusters included in a model (18).

It must also be acknowledged that risk adjustment can be misused. States, managed care organizations, and providers could potentially employ this information as an excuse for poor outcomes. Used appropriately, however, risk adjustment can instead be used to help identify those relatively unchangeable conditions or characteristics of beneficiaries that require the need for additional supports or services if positive HCBS outcomes are to be achieved.

Despite these considerations, the use of risk adjustment techniques in assessing outcomes related to HCBS warrants exploration. Herman et al. (19) in an meta-analysis of risk adjusters found that diagnostic (e.g., illness severity) and demographic (e.g., age) risk adjusters accounted for 6.7% of variance on average and up to 22.8% in models maximizing the use of adjusters. These findings clearly indicate that risk adjustment can make a significant difference in results and their interpretation.

### Study Purpose

This study sought to identify and assess individual level risk adjusters of HCBS outcomes for people with several different types of disabilities. In order to reduce complexity and because the majority of the studies identified included individual-level factors, systems-level risk adjusters are not a focus of this article. Study efforts first focused on the identification, cataloging, and evaluation of risk adjusters used in current research within HCBS. A set of risk adjusters common across disability populations was then used in the data collection process. In the final step of the process, the relevance of risk adjusters in relation to HCBS outcomes for people with disabilities, as specified in the domains and subdomains of the NQF's (20) conceptual framework, were reviewed. Based on the study purpose, the following research questions were addressed:

1. What is the population of existing individual risk adjusters used in examining the outcomes of people with disabilities who are HCBS beneficiaries and the frequency of their use?
2. What is relative importance of existing individual risk adjusters in HCBS outcome measurement as determined by experts in the field?
3. With what frequency have the reviewed risk adjusters been used to better understand outcomes in NQF HCBS measurement domains experienced by people with different types of disabilities?

## Method

Although a systematic review of the literature is a step toward creating a more reliable scientific-basis for confirming or refuting ideas about the use of risk adjusters in disability related-outcome research, this approach suffers from several shortcomings (21). In and of themselves, such reviews do not utilize a systematic tool for combining the results of multiple studies and lack methods necessary to merge findings together to provide a more reliable understanding of outcomes. Their focus is often on statistical significance rather than the magnitude of effects and rarely are critical factors including sample characteristics and study design features factored into outcome evaluations.

While a systematic review aims to provide a comprehensive literature search with pre-defined eligibility criteria, a *meta-analysis* combines and synthesizes findings with statistical models (22). In doing so it statistically assesses effect sizes and models the effect sizes with study characteristics focusing on the magnitude of the effect size (23, 24). Effect sizes are weighed by their precision and in addition to the ability to determine average effect size, one can also estimate the consistency of effects across different studies. The approach also lends itself to the use of moderators to explain observed variations in effect size.

However, a meta-analysis is not always the best solution to understand the impact of one set of variables on others. Research extracted from the extant literature may not include sufficient information to calculate the effect sizes needed for a meta-analysis. A more elementary question is whether there are a sufficient number of primary investigations for a valid meta-analysis to be undertaken in the first place. Although it is theoretically possible to conduct a meta-analysis with only a few studies, drawing conclusions on the basis of small, less than robust and representative samples is likely to lead to unstable results. Another potential reason for using a methodology other than meta-analysis is when the existing research consists of studies with decidedly different objectives, designs, measures, and samples that make it conceptually difficult to combine studies (25). The availability of only a small number of well-conducted empirical studies with representative samples may also indicate that the field is not mature enough to yield useful findings utilizing this approach.

Although there has been a considerable amount of work undertaken in order to better understand the impact of HCBS on beneficiaries, well-designed studies that have utilized risk adjustment remain limited. For this reason, as well as limitations of the existing research in relation to requirements for valid meta-analysis, it would be pre-mature at this time to utilize this approach. As described in the following section, as an alternative we employed a process believed to be more appropriate for the current state of the field. It entailed: (1) the identification of risk adjusters in published HCBS outcome research and their frequency of use; (2) ratings of the importance of the risk adjusters that have be used in HCBS outcome research by technical expert panels; and (3) assessment of the extent to which these risk adjusters have been used to explain HCBS outcomes as specified by NQF HCBS outcome domains (20).

### Literature Search and Study Selection

The first phase of the study consisted of a systematic review of literature on potential risk adjusters associated with the outcomes experienced by HCBS beneficiaries with five different types of disability including intellectual and developmental disability, physical disability, psychiatric disability, age-related disability, and traumatic brain injury. We worked within our project team and technical experts to develop a comprehensive list of keywords. Keywords included combinations of the following words and phrases as Boolean operators: quality of life, outcome, community, risk adjustment, risk factor, covariate, disability, intellectual disability (ID), developmental disability (DD), intellectual and developmental disability (IDD), aged, residential support, independent living, transition, Assertive Community Treatment (ACT), mental health, HCBS, physical disability, traumatic brain injury (TBI), mobility disability.

Searches were conducted across disciplines and research areas, including disability, mental health, social work, gerontology, policy, and public health using the following databases: the Cochrane Database of Systematic Reviews, Google Scholar, JSTOR, Academic Search Premier, PsychINFO, Social Work Abstracts, The National Rehabilitation Information Center, CINAHL, Ovid Medline, Social Sciences Citation Index (Web of Science), and PubMed. In addition, the most recently published research was directly reviewed in a variety of disability-focused journals including the American Journal on Intellectual and Developmental Disabilities, Disability and Health Journal, Disability Studies Quarterly, Journal of Community Health, Journal of Community Practice, Journal of Mental Health, Journal of Healthcare for the Poor and Underserved, Journal of Aging and Health, Journal of the American Geriatrics Society, Journal of Aging and Social Policy, Social Work Research, the Gerontologist, the Journals of Gerontology Series B, and Journal of Gerontological Social Work. Using the keywords listed above in the stated databases resulted in an initial set of 263 studies.

Two sets of evaluative criteria to identify high-quality studies were applied. The first seven criteria listed below are based on previously established standards (26). To these criteria three additional standards developed by our research team: recency (i.e., studies published after 2000), quantitative data and statistical analyses used, and samples with adults with disabilities (i.e., individuals ages 18 and above) were added.

**Figure 2:**
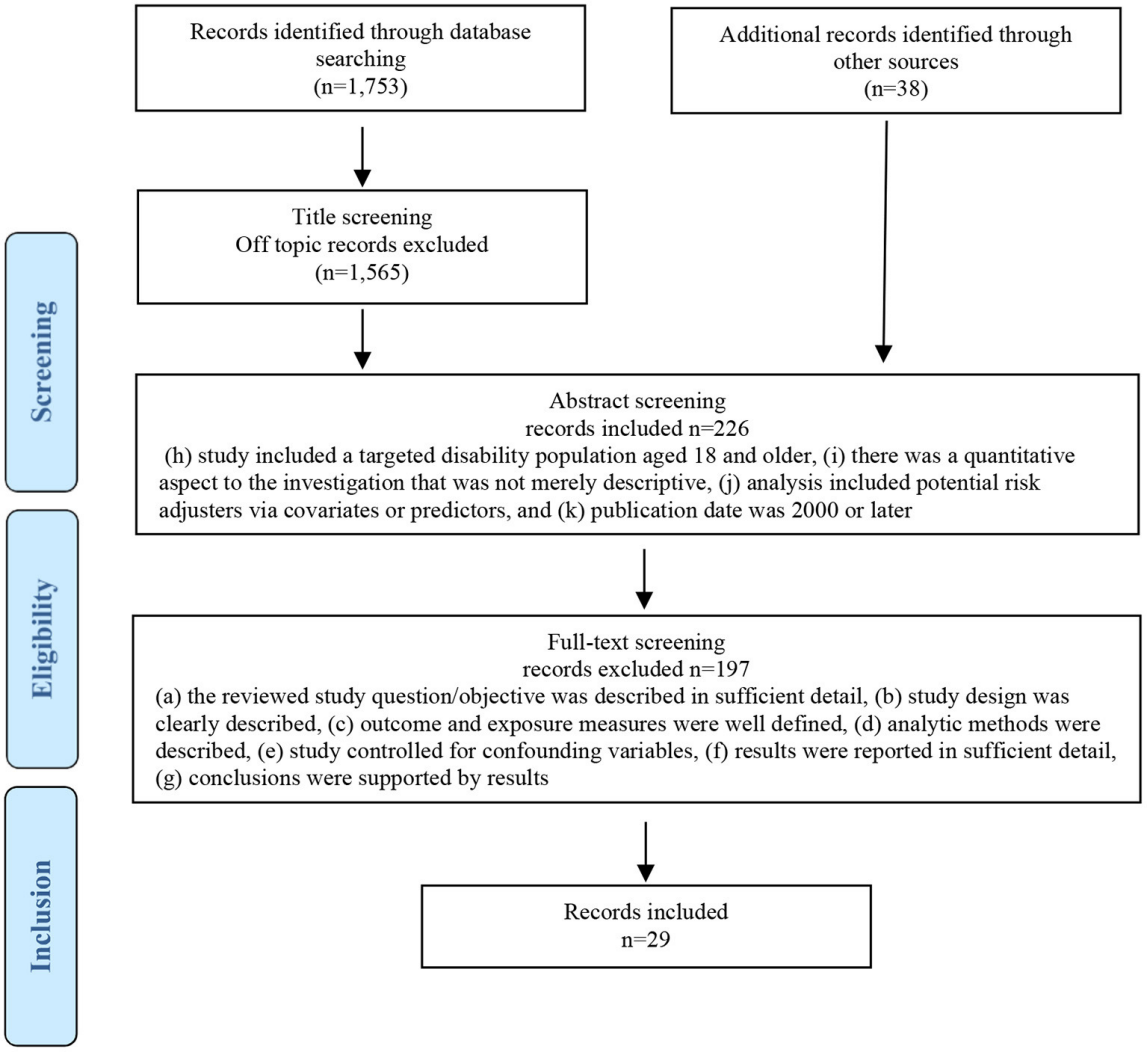
Flow diagram of literature search and selection process.

The final selection criteria required that: (a) the reviewed study question/objective was described in sufficient detail, (b) study design was clearly described, (c) outcome and exposure measures were well defined, (d) analytic methods were described, (e) study controlled for confounding variables, (f) results were reported in sufficient detail, (g) conclusions were supported by results, (h) study included a targeted disability population aged 18 and older, (i) there was a quantitative aspect to the investigation that was not merely descriptive, (j) analysis included potential risk adjusters via covariates or predictors, and (k) publication date was 2000 or later. After applying these criteria to the initial set of 263 studies, a final dataset of 29 investigations focused on HCBS outcomes associated with the NQF's (20) conceptual Framework for HCBS Outcome Measurement met inclusionary criteria for the present study. Figure 2 provides more detail in our selection process.

### Identification of Risk Adjusters

Each study that met inclusion criteria was assigned a unique ID and coded by two coders who kept detailed notes for each risk adjuster identified. In addition, measurement instrument(s) used, response options/scale of measurement, and target population were coded. Following the selection of that portion of the research literature that met the required criteria, all variables included in statistical models within each of the selected articles were independently identified and cataloged by two coders. These variables included all predictors, covariates, and outcomes. Table 1 shows a snapshot of the cataloging process. The two coders met weekly with a third team member to compare their coding and confirm that each variable had been entered correctly. Any discrepancies between coders were discussed. The final decision regarding risk adjuster inclusion or exclusion was achieved by consensus between all three researchers.

**Table 1:** Process of cataloging studies and variables.

**Study ID**	**Study author and year**	**Include (y/n)**	**Population**	**Citation**	**Predictors (coder 1)**	**Predictors (coder 2)**	**Predictors (decision based on agreement)**	**Outcome (coder 1)**	**Outcome (coder 2)**	**Outcome (decision based on agreement)**	**Notes**
1	Jones (2019)	Yes	TBI	APA	Names, measures	Names, measures	Names, measures	Names, measures	Names, measures	Names, measures	

Using this method, variables were extracted from all studies in the data corpus. Coders identified and listed all variables included in each study, sorted them as either *potential* risk adjusters (predictors and undefined covariates that could be risk adjusters) or outcome variables and entered them into the study database. Variables were coded as outcomes if they were identified by the study author(s) as such and included in a statistical model as an outcome. All variables that were not outcomes included in a statistical model as having a statistically significant relationship with an outcome were coded as potential risk adjusters^1^.

### Research Question #1: Coding of Risk Adjusters

Once potential risk adjusters had been entered into the database, they were reviewed and grouped into categories based on their concept of focus and characteristics. Categories are higher order concepts that subsume lower-order concepts that share observations or properties (27, 28). In our case, categories were developed by grouping together shared concepts or characteristics. For example, several studies included depression as a risk adjuster. Although these studies used various instruments to measure depression, the intent to control for depression was the same across studies. Therefore, although depression was measured differently across studies, the risk adjuster that each study identified was the same, and consequently each risk adjuster was labeled using the same code. Using this method, the team grouped all extracted risk adjusters into categories.

New codes and definitions for groups of risk adjusters were developed and updated by coders, a third tiebreaker, and with assistance from the research team at weekly meetings. Any risk adjuster that did not fit an existing category was discussed at weekly meetings with the research team and reviewed to determine whether a new code was justified. Once the codebook was updated to accommodate any new information, final decisions for codes to be applied were determined by consensus. Each risk adjuster received a single code based on the final version of the codebook. The formation of “new” categories ceased at a point at which saturation was reached and new risk adjuster types were not being found. Table 2 shows the definitions for the final group of risk adjusters developed for the purposes of this study. Once this process was complete, frequency counts could take place indicating the number of eligible studies in which each identified risk adjuster was employed.

**Table 2:** Internal definitions used to categorize risk adjusters.

**Risk adjuster**	**Definition**
Age	Age in years or age range
Caregiver characteristics	Features of non-staff caregivers relevant to their care of persons with disabilities
Chronic conditions	Presence of long-term physical conditions which may have implications for mortality
Cognition	Current intellectual functioning, including the ability to remember, recall, learn, concentrate, or make decisions
Comorbidity	Co-occurrence of more than one physical and/or mental health-related condition in the same person, simultaneously or sequentially, where one condition may be primary and another secondary
Condition duration	Duration of time since the onset of a condition, injury or change in health status which led to physical or mental health symptoms
Education	Level of or number of years of schooling
Employment	Current employment status and/or type of employment
Ethnicity/Race	Social group with a common national or cultural tradition
Family member demographics	Information about members of the participant's family, such as parents or siblings
Formal supports	Type or amount of support received from care provider networks, government, or organizations—availability, specificity, satisfaction, and overall degree of care received
Functional disability	Level of functionality in daily life in the presence of short or long-term limitations due to a disabling condition or health problem
Health Indicators	Any physiological measure known to predict health outcomes, specifically decreased functionality and morbidity
Income	Amount of money regularly received by household, family, or individual
Length of stay	Duration of stay at a hospital, rehab, or inpatient care facility
Living arrangement	Type of residence in which the individual lives, including the type of facility and with whom they live
Mental health	Indicators of mental health functioning, including mental health diagnoses
Number of children	How many people, usually children, the individual helps take care of or has living in their household
Population type	Membership in a specific disability population
Region	Geographic location
Relationship status	Whether the individual currently has a partner, a spouse, or are not currently in a relationship
Risky behaviors	Engagement in risky or negative behaviors that have implications for the development of health-related conditions or socially undesirable outcomes
Self-efficacy	The individual believes that he or she can engage in the behaviors necessary to exercise choice and control over aspects of their lives
Sex/Gender	Sex or gender of individual, typically self-reported
Social support availability/engagement	Type or amount of support received, either by availability or by choice, from informal or socially close sources such as family, friends, or community
Symptom severity	The degree of physical, mental health, or cognitive symptoms experienced or change in functional status within a given time interval
Use of a proxy	The degree to which the person with a disability answers questions relevant to their care as opposed to some other individual such as a parent or staff member

### Research Question #2: Expert and Internal Ratings of Risk Adjusters

#### Expert Panel Ratings

The number of times a risk adjuster is included in the literature is not necessarily an indication of its quality. The second phase of this study therefore entailed the convening of TEPs to evaluate the importance, feasibility, and usefulness of the risk adjusters found in the literature.

For our expert panels, we collaborated with scholars in the field of disability and HCBS, including representatives from the field of intellectual and developmental disabilities, traumatic brain injury, physical, psychiatric and age-related disabilities. Technical experts (n = 16) were selected from a variety of sources including the leadership committee for the Rehabilitation Research and Training Center on HCBS Outcome Measurement, The National Advisory Committee of the Center, provider agencies, The Association of University Centers on Disability, and state HCBS programs. Members from university settings and/or research centers affiliated with universities were selected not only due to their knowledge of HCBS but on the basis of their work with target populations. The experience of panel members in HCBS and their disability-specific fields ranged from 14 to 35 years. Each TEP member expert was asked to independently rate the quality of the HCBS relevant risk adjusters identified in the literature.

TEP ratings were obtained using the Qualtrics survey platform. Panel members were asked to prioritize which risk adjusters should be included in future research by independently rating them on multiple dimensions (described below) which were submitted to and agreed upon by the TEP prior to administration of the survey.

TEP members rated the priority of each risk adjuster by independently considering its: (1) feasibility, (2) usability, (3) importance, and (4) accuracy. For this study, *feasibility* was operationally defined as the extent to which information for risk adjustment could be captured without undue burden to participants and/or those collecting and using the data. *Usability* was conceptualized as the likelihood that information provided by the risk adjuster in question would be understandable and useful to its intended audience and lead to either quality improvement and better decision making in HCBS. *Importance* was defined as the relevance of the risk adjuster to the lives of HCBS beneficiaries and how likely the risk adjuster would be helpful in explaining outcomes in multiple domains. *Accuracy* was conceptualized as the ability of the risk adjuster to reliably provide the valid information over time and across data sources.

After the initial rating by TEPs, data were compiled and presented to panels for discussion. Based on the feedback provided, operational definitions and dimensions of quality (feasibility, usability, importance, and accuracy) were modified and a final version of the survey re-submitted. This revised survey included suggested new risk adjusters and improved operational definitions. After administering this survey, data were summarized and presented back to the panel. Summary data consisted of frequency counts, mean ratings, and standard deviation calculations to determine the average importance and level of agreement for each risk adjuster. After reviewing results, TEPs engaged in discussion and worked toward achieving consensus on a final ranked list of risk adjusters and definitions. In this way, widely-used risk adjusters viewed by TEP members as most critical to develop a better understanding of the outcomes experienced by HCBS beneficiaries were identified.

#### Internal Ratings With Respect to Target Populations and Outcomes

Following the input of TEP members, an internal panel of research staff independently assessed the selected risk adjusters in terms of their suitability for use with different populations and outcomes. These ratings were undertaken to control for the difference between general covariates (or predictor variables) and variables better suited for risk adjustment (NQF). Utilizing the guidelines provided by the NQF [(7), pp. 9–10] each rater independently assessed the list of risk adjusters. These ratings, in conjunction with TEP evaluation were used in order to: (1) clarify differences between risk adjusters and other covariates, and (2) identify risk adjusters that would be appropriate for use with multiple disability groups and HCBS-related outcomes and to further guide our recommendation process.

### Research Question #3: Risk Adjusters and Study Outcomes

A third round of coding was utilized to classify outcomes included in the reviewed studies into conceptual groupings and determine their correspondence with NQF HCBS outcome domains (20) as well as organize outcomes into interpretable categories for analysis. This process entailed two coders independently coding each outcome into NQF outcome domains, plus two additional domains (employment and transportation) identified as of critical importance by stakeholder groups. Following initial coding, the two coders met with a third researcher who served as a mediator and ultimate tiebreaker with respect to outcomes in which there was disagreement between raters.

## Results

### Research Question #1: Summary of Risk Adjusters by Populations

In order to address the first research question and determine the distribution of existing quantitative risk adjusters used in disability-related research associated with the NQF's (20) HCBS Outcome Measurement Framework, we calculated frequencies both for the overall use of each risk adjuster and separately for each targeted disability group (see Table 3).

**Table 3:** Individual-level risk adjusters by population group.

**Risk adjuster**	**IDD**	**MH**	**Older adult**	**PD**	**TBI**	**Total count**	**Total % of RAs**
Functional disability	25	5	60	16	20	126	15.8
Chronic conditions	17	2	72	20	21	132	16.5
Age	10	4	26	7	8	55	6.9
Mental health	8	4	32	8	9	61	7.6
Cognition	9	0	28	7	11	55	6.9
Sex/gender	7	2	24	5	9	47	5.9
Depression	2	2	17	3	7	31	3.9
Ethnicity/race	1	1	22	1	4	29	3.6
Education	3	2	13	4	6	28	3.5
Health indicators	2	1	11	5	4	23	2.9
Social support availability/engagement	5	2	9	0	6	22	2.8
Formal supports	3	2	7	0	9	21	2.6
Risky behaviors	7	0	10	4	6	27	3.4
Relationship status	1	2	12	0	5	20	2.5
Income	2	1	10	2	4	19	2.4
Living arrangement	4	1	12	0	0	17	2.1
Region	3	0	12	0	1	16	2.0
Symptom severity	0	0	2	4	7	13	1.6
Length of stay	1	0	10	1	1	13	1.6
Employment	4	1	0	1	6	12	1.5
Condition duration	1	0	2	2	5	10	1.3
Comorbidity	0	0	6	0	0	6	0.8
Population type	3	0	1	1	0	5	0.6
Family member demographics	2	0	0	0	1	3	0.4
Self-efficacy	1	0	1	1	0	3	0.4
Use of a proxy	1	0	2	0	0	3	0.4
Caregiver characteristics	0	0	0	0	1	1	0.1
Number of children	0	1	0	0	0	1	0.1
Total count	122	33	401	92	151	799	
Total % by disability group	15.3	4.1	50.2	11.5	18.9		

*IDD, intellectual and developmental disability; MH, mental health; PD, physical disability; TBI, traumatic brain injury*.

The most commonly included individual-level risk adjusters were *functional disability* and *chronic conditions*, whereas the least common risk adjusters were caregiver characteristics and number of children. Demographic risk factors, such as age, sex/gender, ethnicity/race, and education level were frequently used as individual risk factors in all likelihood due to their accessibility and known ability to predict variance in outcomes. Physical and mental health-related risk adjusters (e.g., mental health diagnoses), risky behavior (e.g., drug use, smoking), and health indicators (e.g., BMI, BP) were also commonly utilized. This finding reflects the medical nature of much of the research on individuals with disabilities and the large number of studies on aging included in our sample. The described individual-level risk adjusters were identified across all population groups with the most frequently targeted population people with age-related disability (*n* = 401; 50% of total studies). People with psychiatric related disability was the least frequently covered population (*n* = 33).

With regard to the variables overlapping with the internal ratings based on NQF risk adjustment guidelines, we note that age, ethnicity/race, and sex/gender have been commonly used in many fields and population type specific to studies targeting multiple disability populations (e.g., aging and IDD groups).

### Research Question #2: Survey Importance Ratings

In order to evaluate the importance, feasibility, and usefulness of existing risk adjusters used in HCBS-related research focused on outcomes included in the NQF framework, a TEP was convened and the ratings of reviewed risk adjusters analyzed. Ratings of risk adjusters by TEP members are shown in Table 4. For brevity and to highlight the top-rated risk adjusters, Table 4 only includes those risk adjusters that were rated in the top half of those found in the literature with respect to importance. Bolded risk adjusters were also rated highly by our internal group of RTC/OM research staff based on NQF guidelines.

**Table 4:** Average ratings by expert panel of top half of rated individual level risk adjusters.

**Risk adjuster**	**Average rating**
Cognition	4.3
**Chronic conditions**	**4.1**
Living arrangement	4.1
Natural support engagement	4.1
Functional disability	4.1
**Age**	**4**
Mental health	3.9
**Population type**	**3.7**
Formal supports and services	3.6
Level of Communication	3.6
**Ethnicity/race**	**3.6**
Income	3.6
**Sex/gender**	**3.6**
**Comorbidity**	**3.6**
Employment	3.6

*Mean rating for all risk adjusters was 3.5 (SD = 0.04). Bolded risk adjusters also ranked highly based on internal rankings of research project staff based on NQF guidelines. The potential range rankings was from 1 to 5*.

As can be seen in Table 3, a variety of *individual characteristics* (e.g., cognitive status, functional disability and age), *contextual factors* (e.g., living arrangement, formal supports and services), and health conditions (e.g., diabetes) were rated as having a high level of relevance. Note that some of these variables can be outcomes themselves, namely income and employment.

### Research Questions #3: Risk Adjusters and Study Outcomes

In order to address our third research question on the alignment of the reviewed risk adjusters with outcomes identified in peer reviewed articles and organized by NQF domains, we calculated the frequency of use of each risk adjuster. This frequency was based on the percentage each risk adjuster was utilized within each NQF domain across identified outcomes (Table 5) provides a summary of these results. Risk adjusters listed in Table 5 are in the same order as in Table 2, from most commonly to least commonly used across reviewed studies.

**Table 5:** Percentages and frequency of risk adjusters used across NQF domains.

**Risk adjusters**	**Multiple domains** **(*N* = 11)**	**Holistic health and functioning** **(*N* = 24)**	**Service delivery and effectiveness** **(*N* = 3)**	**Community inclusion** **(*N* = 4)**	**Choice and control** **(*N* = 2)**	**Human and legal rights** **(*N* = 1)**	**Caregiver support** **(*N* = 1)**	**Employment** **(*N* = 2)**
Functional disability	63.6% (7)	58.3% (14)	66.7% (2)	75.0% (3)	100.0% (2)	100.0% (1)	0.0% (0)	50.0% (1)
Chronic conditions	27.3% (3)	37.5% (9)	66.7% (2)	25.0% (1)	50.0% (1)	100.0% (1)	0.0% (0)	0.0% (0)
Age	54.5% (6)	95.8% (23)	100.0% (3)	75.0% (3)	100.0% (2)	100.0% (1)	0.0% (0)	50.0% (1)
Mental health	45.5% (5)	29.2% (7)	33.3% (1)	50.0% (2)	100.0% (2)	100.0% (1)	100.0% (1)	0.0% (0)
Cognition	27.3% (3)	41.7% (10)	66.7% (2)	50.0% (2)	50.0% (1)	100.0% (1)	100.0% (1)	0.0% (0)
Sex/gender	54.5% (6)	83.3% (20)	66.7% (2)	50.0% (2)	50.0% (1)	100.0% (1)	0.0% (0)	50.0% (1)
Depression	27.3% (3)	41.7% (10)	33.3% (1)	50.0% (2)	50.0% (1)	100.0% (1)	0.0% (0)	0.0% (0)
Ethnicity/race	9.1% (1)	58.3% (14)	33.3% (1)	0.0% (0)	0.0% (0)	0.0% (0)	0.0% (0)	0.0% (0)
Education	9.1% (1)	54.2% (13)	33.3% (1)	50.0% (2)	0.0% (0)	0.0% (0)	0.0% (0)	50.0% (1)
Health indicators	9.1% (1)	20.8% (5)	33.3% (1)	0.0% (0)	0.0% (0)	0.0% (0)	0.0% (0)	0.0% (0)
Social support availability/engagement	27.3% (3)	12.5% (3)	0.0% (0)	0.0% (0)	0.0% (0)	0.0% (0)	0.0% (0)	50.0% (1)
Formal supports	0.0% (0)	20.8% (5)	100.0% (3)	0.0% (0)	0.0% (0)	0.0% (0)	0.0% (0)	50.0% (1)
Risky behaviors	0.0% (0)	20.8% (5)	0.0% (0)	0.0% (0)	50.0% (1)	0.0% (0)	0.0% (0)	0.0% (0)
Relationship status	9.1% (1)	33.3% (8)	66.7% (2)	25.0% (1)	0.0% (0)	0.0% (0)	0.0% (0)	50.0% (1)
Income	9.1% (1)	25.0% (6)	0.0% (0)	25.0% (1)	0.0% (0)	0.0% (0)	0.0% (0)	0.0% (0)
Living arrangement	9.1% (1)	29.2% (7)	33.3% (1)	0.0% (0)	50.0% (1)	0.0% (0)	0.0% (0)	0.0% (0)
Region	0.0% (0)	8.3% (2)	33.3% (1)	0.0% (0)	50.0% (1)	0.0% (0)	0.0% (0)	0.0% (0)
Symptom severity	27.3% (3)	12.5% (3)	33.3% (1)	25.0% (1)	0.0% (0)	0.0% (0)	0.0% (0)	50.0% (1)
Length of stay	45.5% (5)	8.3% (2)	0.0% (0)	25.0% (1)	50.0% (1)	100.0% (1)	0.0% (0)	0.0% (0)
Employment	9.1% (1)	16.7% (4)	0.0% (0)	25.0% (1)	0.0% (0)	0.0% (0)	0.0% (0)	50.0% (1)
Condition duration	27.3% (3)	16.7% (4)	0.0% (0)	25.0% (1)	0.0% (0)	0.0% (0)	100.0% (1)	0.0% (0)
Comorbidity	0.0% (0)	16.7% (4)	0.0% (0)	0.0% (0)	0.0% (0)	0.0% (0)	0.0% (0)	0.0% (0)
Population type	9.1% (1)	4.2% (1)	0.0% (0)	0.0% (0)	50.0% (1)	0.0% (0)	0.0% (0)	0.0% (0)
Family member demographics	0.0% (0)	8.3% (2)	0.0% (0)	0.0% (0)	0.0% (0)	0.0% (0)	0.0% (0)	0.0% (0)
Self-efficacy	0.0% (0)	8.3% (2)	0.0% (0)	0.0% (0)	0.0% (0)	0.0% (0)	0.0% (0)	0.0% (0)
Use of a proxy	0.0% (0)	0.0% (0)	0.0% (0)	0.0% (0)	50.0% (1)	0.0% (0)	0.0% (0)	0.0% (0)
Caregiver characteristics	0.0% (0)	0.0% (0)	0.0% (0)	0.0% (0)	0.0% (0)	0.0% (0)	0.0% (0)	0.0% (0)
Number of children	0.0% (0)	4.2% (1)	0.0% (0)	0.0% (0)	0.0% (0)	0.0% (0)	0.0% (0)	0.0% (0)

*Numbers is brackets () denote the frequency of studies. Risk adjusters listed same order as Table 2, from most commonly used to least commonly used across studies assessed. The NQF domain “System Performance and Accountability” is not included as it was not an outcome in the studies assessed in this analysis. Ns per domain here exceed the 29 studies that are mentioned in the methods section as meeting inclusion criteria due to many studies having included more than 1 outcome included separately in this analysis*.

As can be seen in Table 5, studies with outcomes in the NQF domain *Holistic Health & Functioning* included the largest number of risk adjusters, followed by those addressing out-comes across *multiple domains* and *Community Inclusion*. The remaining NQF domains were found to have four or less risk adjusters. NQF domains not included in Table 5 were found to have no studies that included the use of risk adjustment. The most frequently included risk adjuster corresponding to the Holistic Health and Functioning domain was age, followed by gender, functional disability and ethnicity/race. Studies with outcomes across multiple domains included functional disability as most frequent risk adjuster, followed by age sex/gender, mental health and length of stay. Functional disability and age were the most frequently used risk adjusters among studies focused on the Community Inclusion domain of the NQF framework.

## Discussion

People with disabilities who receive HCBS experience a variety of outcomes based on their disability and the quantity and quality of services they receive. In the US, presence or absence of needed services, the quality of these supports, and their match to the person's needs vary dramatically between states, regions, and cities (29). When states or agencies evaluate the effectiveness of HCBS these efforts typical focus on directly examining the personal outcomes an individual experiences with covariates sometimes used to control for confounding factors. Unlike the healthcare field, risk adjustment has not been routinely used in HCBS evaluations to adjust results. The need for such adjustment is a result of inequalities that may exist as a result of gender, type and level of disability, quality, type, and intensity of supports, etc. at an individual, organization, or state level. Failure to take such differences into account when interpreting results can lead to an exacerbation of service and outcome inequities.

The purpose of this study was to contribute to the efforts toward making evaluations of service provision more accurate and equitable by conducting a systematic review and expert ratings of using risk adjusters in HCBS. The study was designed to: (a) identify, catalog, and evaluate risk adjusters used in recent HCBS-related research; (b) prioritize a set of risk adjusters that are useful in HCBS-related research across disability populations and, (c) identify the types of risk adjusters used in studies focused on various NQF domains.

Based on the results of this study, we recommend that four primary risk adjusters be tested in future investigations for use in HCBS outcome measurement. These risk adjusters include: (1) *chronic conditions* (presence of long-term physical conditions that may have implications for mortality); (2) *functional disability* (level of functionality in daily life in the presence of short or long-term limitations due to a disabling condition or health problem); (3) *mental health* (indicators of mental health functioning, including mental health diagnoses); and (4) *cognition* (current intellectual functioning, including the ability to remember, recall, learn, concentrate, or make decisions). These four risk adjusters have been recommended for future testing because they: are not specific to a particular disability population, were rated highly by members of TEPs, matched recommended NQF guidelines for risk adjusters, and included under at least two NQF domains. We excluded demographic risk adjusters from our recommendations since they are commonly used as covariates and we wish to highlight other factors with potentially confounding effects when studying particular outcomes.

The recommendation presented are consistent with the findings of several previous investigations that identified similar factors that control for confounding variables in explaining HCBS-related outcomes. Based on their analyses, the National Core Indicators—Aging and Disabilities (NCI-AD) data collection program, for example, has identified 15 characteristics they recommend as risk adjusters including: the amount of assistance needed for everyday activities and for self-care, overall health, level of hearing, level of vision, presence of a mental health diagnosis, and whether a person forgets things (30). Tichá et al. (31) in the summary findings of predictors based on studies using the NCI – In Person Survey (NCI-IPS) found that at the individual level, challenging behavior, psychiatric diagnosis, and level of ID, had significant explanatory power in accounting for outcomes among people with IDD. Herman et al. (19) identified severity of diagnosis, substance abuse, baseline functioning and quality of life as significant factors in clinical outcomes in their review of literature of risk-adjusting outcomes of mental health and substance-related care. The result of these studies support the four constructs put forth as recommended risk adjusters for further study.

### Prioritized Risk Adjusters

This next section summarizes information on the recommended risk adjusters of chronic conditions, functional disability, mental health and cognition based on previous research within the context of risk adjustment in HCBS.

#### Chronic Conditions

A chronic condition is a persistent or otherwise long-lasting human health condition or disease that lasts for more than 3 months. In the US, 25% of adults have at least two chronic conditions (32). Two frameworks conceptualizing the effect of chronic conditions on disability are the *Disablement Process* (33) and the *International Classification of Functioning, Disability, and Health* (34). Both frameworks posit that health conditions intersect with the environment to lead to activity limitations (35). This intersection is exactly how chronic conditions function as potential risk adjusters in disability research. Some disabilities can be considered primary chronic conditions (e.g., arthritis), while others (e.g., mild arthritis) experienced by a person with IDD could be considered a secondary condition. The accumulation of such conditions among members of a population can make it difficult to disentangle the differences between service delivery approaches when conditions are not evenly distributed between individuals or groups. Research has demonstrated that limitations in role performance due to chronic conditions (e.g., diabetes etc.) can lead to difficulties in performing valued activities (36). Thus, when assessing outcomes, risk adjusting for the prevalence of chronic conditions may capture variance unrelated to the primary disability of interest, thereby improving estimates of other factors (e.g., an intervention) being studied.

#### Functional Disability

Functional disability has been conceptualized as one's functional status, capacity, limitations, and/or disability status (37, 38) and has been used to better understand physical frailty, fatigue, Activities of Daily Living (ADLs), Instrumental Activities of Daily Living (IADLs), and mobility (39, 40). Currently, however, there is not a uniform definition of functional disability, with each set investigators drawing conclusions based on their own, personal perspectives on the construct.

Risk-adjustment may not always be meaningful with respect to functional disability as it can vary from a long-term limitations to those that are episodic or co-occurring along with other conditions. For example, whereas physical frailty was found to be associated with risk of mild cognitive impairment (39), cause and effect are not known (41). The use of functional disability as a risk adjuster therefore depends on whether the assumed type of a functional disability is a stable trait. For some sub-populations (e.g., people with TBI or age-related disabilities) functional ability is likely to change over time. For others (e.g., people with IDD) this is not as likely to be the case. Thus, risk-adjusting for the IDD sub-population makes sense whereas it does not for persons with TBI or age-related disability.

#### Mental Health

The World Health Organization (WHO) defines mental health as a state of well-being in which people realize their own abilities, can cope with the stresses of everyday life, work productively, and are able to make contributions to their communities (34). People with disabilities often experience disadvantage that contributes to poor mental health (e.g., poverty, etc.) (42–44).

Although studies have attempted to appraise mental health in persons with disabilities, this has been challenging due to the heterogeneity among studies with respect to samples, range and type of disability, and mental health interventions under study (45). In addition, the scientific quality of studies at times falls short in terms of incomplete reporting of analyses, lack of clear definitional criteria, and the risk of bias (45, 46). These limitations as well as the myriad of formal and informal factors potentially contributing to mental health outcomes have, thus far, precluded establishing robust evidence as to risk adjusters that would likely generalize to all groups with disabilities.

Risk-adjustment for mental health also requires accounting for both type and severity of mental health conditions as manifested in various disability groups. For example, risk-adjusting for depression and/or anxiety due to congenital disability (e.g., cerebral palsy) may make more sense than adjusting for time-limited depression associated with an injury or stress that can be alleviated by adjustments in the environment.

#### Cognition

Cognition refers to the mental processes involved in gaining, retaining, and effectively using knowledge to adapt to ones' environment. Cognitive processes include thinking, knowing, remembering, judging, and problem-solving (APA Dictionary of Psychology, 2018). They are higher-level functions that include language, imagination, perception, and planning and can be measured in terms of both cognitive ability and disability. Cognitive disability has been associated with greater risk for less self-efficacy (42), and lower quality of life (47) not only among people with IDD but those with age-related and/or physical disabilities. It is related to the duration a person with age-related disability spend in hospital-level care (48, 49) and has been shown to mediate the relationship between physical activity and lower blood pressure (50). Among individuals with IDD, level of intellectual disability, a measure directly related to cognitive ability, has been associated with lower levels of self-determination (51), less choice-making (12) and lower levels of community-based employment (52–55).

The use of cognition (ability or disability) as a risk adjuster has the potential to help control for a significant amount of variation in outcomes within and between different disability groups. Considering cognition as a risk adjuster can potentially increase the accuracy of comparisons between groups on these outcomes. However, researchers must consider that some groups (e.g., people with age-related disabilities and TBI) can experience significant natural decreases or increases in cognitive capacities over extended periods while others (e.g. people with psychiatric disability) may demonstrate fluctuating capacity over periods as short as a day. Both of these situations can have a significant impact on the reliability of results.

## Conclusion

The main aim of this study was to review and evaluate risk adjusters currently used in disability-related outcome research. Based on the findings, we have provided recommendations for potential risk adjusters that would appear to merit further empirical investigation that are: (a) not specific to a particular disability population, (b) rated highly by expert panels, (c) matched to the suggested NQF guidelines for risk adjusters, and (d) included under at least two NQF HCBS outcome domains. Based on what we have learned we can conclude that:

1. Demographic characteristics (e.g., gender, race, education level) are the most commonly used risk adjusters suggesting that both their known associations and feasibility are strong factors to consider in risk-adjustment selection. Such risk adjusters can be appropriate to control for unmalleable characteristics, which in turn can increase accuracy of conclusions and comparisons within HCBS.
2. Risk adjustment has most commonly been used in research related to health outcomes. Based upon existing investigations as well as ratings of our TEPs, chronic conditions, functional capacities, mental health condition, and cognition would appear to have the potential to be useful as risk adjusters in models assessing the outcomes experienced by individuals with disabilities. Through the consideration of risk adjustment at the individual level within HCBS, it is likely that the precision with which we are able to match services and supports to the needs of individuals with disabilities and thus improve their outcomes, will increase.

## Future Directions

### Outcome and Population Specificity

Risk adjustment is outcome dependent. Future research is needed to explore the impact of the recommended risk adjusters within the context of specific outcomes and populations within HCBS. Most of the risk adjusters considered in this study were associated with the NQF's Holistic Health and Function domain with few using risk adjustment found to address Human and Legal Rights, Caregiver Support, and Employment; and no risk adjusted observed with respect to Person-Centered Planning and Coordination, Equity, Workforce, or Consumer Leadership in System Development.

In a similar manner, it cannot be expected that a risk adjuster that works effectively with one population with specific levels of support needs will necessarily work well with others. The recommended risk adjusters need to be tested with different populations based on their disability type and intensity of support needs, age, gender, race, etc. to determine whether they function in ways intended and expected.

### Level of Risk Adjustment

Although the purpose of this manuscript was to review broadly the state of risk adjustment in the field of disability research and HCBS, we focused on identifying potential risk adjusters at the level of the individual. There are, however, also factors beyond the individual (e.g., at the systems level) that need to be considered when evaluating HCBS outcomes. Such factors include, but are not limited to, available residential opportunities for people with disabilities, levels of support funding, expenditures of supports and services as well as employment policies and availability in different states or regions. Future research should therefore focus of a review and evaluation of system-level outcomes within HCBS.

## Limitations

An extensive search of the literature was undertaken as part of this study, using specific key words and databases to locate studies that utilized risk adjustment. This approach could have led to leaving out relevant studies. In addition, the process used to code risk adjusters used in the existing research associated with various NQF outcome domains and subdomains ended when project staff used specified criteria to make a determination that construct saturation had been achieved. This approach could also have inadvertently led to leaving out relevant studies and risk adjusters.

Another limitation relates to the operational definition used for the construct of “risk adjuster.” The difference between a covariate (an *explored* variable in relation to an outcome) and a risk adjuster (an intentionally *controlled* variable in relation to an outcome intended to improve the estimation of relationships in a model) is by no means clearly defined within HCBS or by researchers who investigate outcomes of people with disabilities.

It must also be noted that the distribution of NQF-relayed HCBS outcomes identified in the studies reviewed were limited. The dominance of studies focused on Holistic Health and Functioning demonstrates the medical/health focus of the literature identified for the purposes of this investigation. The lack of studied outcomes that fell into other NQF domains and subdomains could be a result of these outcomes being more often treated as mediators or moderators of health outcomes as opposed to important in their own right. These non-medical and non-service constructs, however, have been identified by the NQF, the University of Minnesota Research and Training Center on HCBS Outcome Measurement as well as a wide variety of stakeholders including people with disabilities themselves as critical and relevant for high quality of life, and should be a focus of study in future research.

## Data Availability Statement

The original contributions presented in the study are included in the article/supplementary material, further inquiries can be directed to the corresponding author/s.

## Author Contributions

JH drafted the initial version of the manuscript, worked on the analyses, and lead the manuscript creation process. TK assisted with the analyses and writing of the manuscript. RT and BA both contributed to the initial conceptualization of the research and contributed to the writing of the manuscript. All authors contributed to the article and approved the submitted version.

## Funding

The development of this manuscript was funded by the National Institute on Disability, Independent Living, and Rehabilitation Research (NIDILRR), federal grant # 90RT5039.

## Conflict of Interest

The authors declare that the research was conducted in the absence of any commercial or financial relationships that could be construed as a potential conflict of interest.

## Publisher's Note

All claims expressed in this article are solely those of the authors and do not necessarily represent those of their affiliated organizations, or those of the publisher, the editors and the reviewers. Any product that may be evaluated in this article, or claim that may be made by its manufacturer, is not guaranteed or endorsed by the publisher.

## Footnotes

^1^The distinction between covariates vs. risk adjusters was therefore not made in the present study, and all variables included in statistical models that were significantly associated with an outcome variable were included as potential risk adjusters for the purposes of this study.

## References

[1] Shaughnessy PW Hittle DF. Overview of Risk Adjustment and Outcome Measures for Home Health Agency OBQI Reports: Highlights of Current Approaches Outline of Planned Enhancements. Denver, CO: Center for Health Services Research, UCHSC (2002). Available online at: https://www.cms.gov/Medicare/Quality-Initiatives-Patient-Assessment-Instruments/HomeHealthQualityInits/downloads/HHQIriskadj.pdf (accessed July 29, 2021).

[2] Gressel JW. Development of a quality ranking model for home health care providers. Health Market Q. (2013) 30:246–62. 10.1080/07359683.2013.814503 23924223

[3] Iezzoni LI. Risk adjustment for performance measurement. In: Performance Measurement for Health System Improvement: Experiences, Challenges and Prospects. Cambridge University Press (2009). p. 251.

[4] Jencks SF. Changing health care practices in Medicare's Health Care Quality Improvement Program. Jt Comm J Qual Improv. (1995) 21:343–47. 10.1016/S1070-3241(16)30160-2 7581738

[5] Iezzoni LI editor. Risk Adjustment for Measuring Health Care Outcomes (Vol. 3). Chicago, IL: Health Administration Press (2003).

[6] Fralich JT Booth M Keith RG. Data Quality and Analysis: Managing and Using Home and Community-based Services Data for Quality Improvement. New Brunswick, NJ: The Community Living Exchange Collaborative at Rutgers Center for State Health Policy and the National Academy for State Health Policy (2006).

[7] National Quality Forum. Risk Adjustment for Socioeconomic Status or Other Sociodemographic Factors. Washington, DC: National Quality Forum (2014). Available online at: https://www.qualityforum.org (accessed July 29, 2021).

[8] National Quality Forum. Current Practices of Testing Social and Functional Status-Related Risk Factors Within Risk Adjustment Models of Performance Measurement. Washington, DC: National Quality Forum (2020). Available online at: https://www.qualityforum.org (accessed July 29, 2021).

[9] Centers for Medicare Medicaid Services Center for Consumer Information Insurance Oversight. HHS-Operated Risk Adjustment Methodology Meeting Discussion Paper. (2016). Available online at: https://www.cms.gov/CCIIO/Resources/Forms-Reports-and-Other-Resources/Downloads/RA-March-31-White-Paper-032416.pdf (accessed July 29, 2021).

[10] Velentgas P Dreyer NA Nourjah P Smith SR Torchia MM editors. Developing a Protocol for Observational Comparative Effectiveness Research: A User's Guide. Rockville, MD: Agency for Healthcare Research Quality (2013). Publication No.: 12(13)-EHC099. https://www.ncbi.nlm.nih.gov/books/NBK126190/pdf/Bookshelf_NBK126190.pdf (accessed July 29, 2021). 23469377

[11] Abery BH Roberts M Nyce A Houseworth J Ticha R. Content Validation of the National Quality Forum's HCBS Outcome Measurement FrameworkL Technical Report #1. Minneapolis, MN: University of Minnesota Rehabilitation Research and Training Center on HCBS Outcome Measurement (2012).

[12] Houseworth J Stancliffe RJ Tichá R. Association of state-level and individual-level factors with choice making of individuals with intellectual and developmental disabilities. Res Dev Disabil. (2018) 83:77–90. 10.1016/j.ridd.2018.08.008 30144747

[13] Wehman P Chan F Ditchman N Kang H. Effect of supported employment on vocational rehabilitation outcomes of transition-age youth with intellectual and developmental disabilities: a case control study. Intellect Dev Disabil. (2014) 52:296–310. 10.1352/1934-9556-52.4.296 25061773

[14] Yamamoto SH Alverson CY. Successful vocational outcomes: a multilevel analysis of self-employment through US vocational rehabilitation agencies. J Vocat Rehabil. (2013) 38:15–27. 10.3233/JVR-120617

[15] Tichá R Lakin KC Larson SA Stancliffe RJ Taub S Engler J. Correlates of everyday choice and support-related choice for 8,892 randomly sampled adults with intellectual and developmental disabilities in 19 states. Intellect Dev Disabil. (2012) 50:486–504. 10.1352/1934-9556-50.06.486 23256691

[16] Altman BM. Disability definitions, models, classification schemes, and applications. In: Albrecht G Seelman KD Bury M editors. Handbook of Disability Studies. Thousand Oaks, CA: Sage (2001). p. 97–122.

[17] Fortinsky RH Fenster JR Judge JO. Medicare and Medicaid home health and Medicaid waiver services for dually eligible older adults: risk factors for use and correlates of expenditures. Gerontologist. (2004) 44:739–49. 10.1093/geront/44.6.739 15611210

[18] Murtaugh CM Peng T Aykan H Maduro G. Risk adjustment and public reporting on home health care. Health Care Financ Rev. (2007) 28:77. 17645157 PMC4194996

[19] Herman WH Ma Y Uwaifo G Haffner S Kahn SE Horton ES. Differences in A1C by race and ethnicity among patients with impaired glucose tolerance in the Diabetes Prevention Program. Diabetes Care. (2007) 30:2453–7. 17536077 10.2337/dc06-2003 PMC2373980

[20] National Quality Forum. Evaluation of the NQF Trial Period for Risk Adjustment for Social Risk Factors. Washington, DC: National Quality Forum (2017). Available online at: https://www.qualityforum.org (accessed July 29, 2021).

[21] Cooper C Selwood A Livingston G. Knowledge, detection, and reporting of abuse by health and social care professionals: a systematic review. Am J Geriatr Psychiatry. (2009) 17:826–38. 10.1097/JGP.0b013e3181b0fa2e 19916205

[22] Cheung M Vijayakumar R. A guide to conducting a meta-analysis. Neuropsychol Rev. (2016) 26:121–8. 10.1007/s11065-016-9319-z 27209412

[23] Pigott T. Advances in Meta-Analysis. New York: Springer Science and Business Media (2012).

[24] Schmidt FL. History and development of the Schmidt–Hunter meta-analysis methods. Res Synth Methods. (2015) 6:232–9. 10.1002/jrsm.1134. 26097187

[25] Cheung K Wang TS Farrokhyar F Roman SA Sosa JA. A meta-analysis of preoperative localization techniques for patients with primary hyperparathyroidism. Ann Surg Oncol. (2012) 19:577–83. 10.1245/s10434-011-1870-5 21710322

[26] Kmet LM Cook LS Lee RC. Standard Quality Assessment Criteria for Evaluating Primary Research Papers from a Variety of Fields. HTA Initiative #13. Edmonton, AB: Alberta Heritage Foundation for Medical Research (AHFMR) (2004).

[27] Creswell JW Hanson WE Clark Plano VL Morales A. Qualitative research designs: Selection and implementation. Couns Psychol. (2007) 35:236–64.

[28] Corbin J Strauss A. Developing concepts in terms of their properties and dimensions. In: Corbin J Strauss A editors. Basics of Qualitative Research: Techniques and Procedures for Developing Grounded Theory. London: Sage (2015). p. 239–268.

[29] Larson SA Eschenbacher HJ Taylor B Pettingell S Anderson L Sowers M. In-Home Residential Long-Term Supports Services for Persons With Intellectual or Developmental Disabilities: Status Trends Through 2017. Minneapolis: University of Minnesota, Research Training Center on Community Living, Institute on Community Integration (2020). Available online at: https://icis.umn.edu/files/aCHyYaFjMi/risp_2017 (accessed December 12, 2019).

[30] NCI-AD. National Core Indicators^®^ 2018–19 Aging and Disability (IPS) National Report. (2019). Retrieved from: https://nci-ad.org/upload/reports/NCI-AD_2017-2018_National_Report_Part_1_FINAL_12-24-19.pdf

[31] Tichá R Hewitt A Nord D Larson S. System and individual outcomes and their predictors in services and support for people with IDD. Intellect Dev Disabil. (2013) 51:298–315. 10.1352/1934-9556-51.5.298 24303819

[32] Ward BW Black LI. State and regional prevalence of diagnosed multiple chronic conditions among adults aged≥ 18 years—United States, 2014. Morbid Mortal Wkly Rep. (2016) 65:735–8. 10.15585/mmwr.mm6529a3 27467707

[33] Institute of Medicine. Retooling for an Aging America: Building the Health Care Workforce. (2008). Retrieved from: http://iom.nationalacademies.org/en/Reports/2008/Retooling-for-an-Aging-America-Building-the-Health-Care-Workforce.aspx 25009893

[34] World Health Organization the World Bank. World Report on Disability. Geneva: World Health Organization (2011). Retrieved from: https://www.who.int/disabilities/world_report/2011/report.pdf?ua=1

[35] Freeman RD. Tic disorders and ADHD: answers from a world-wide clinical dataset on Tourette syndrome. Eur Child Adolesc Psychiatry. (2007)16:15–23. 17665279 10.1007/s00787-007-1003-7

[36] Verbrugge LM Patrick DL. Seven chronic conditions: their impact on US adults' activity levels and use of medical services. Am J Public Health. (1995) 85:173–82. 10.2105/AJPH.85.2.173 7856776 PMC1615320

[37] Gulley SP Rasch EK Chan L. The complex web of health: relationships among chronic conditions, disability, and health services. Public Health Rep. (2011) 126:495–507. 10.1177/003335491112600406 21800744 PMC3115209

[38] Keysor JJ Jette AM LaValley MP Lewis CE Torner JC Nevitt MC. Community environmental factors are associated with disability in older adults with functional limitations: the MOST study. J Gerontol Ser A: Biomed Sci Med Sci. (2010) 65:393–399. 10.1093/gerona/glp182 19995830 PMC2905834

[39] Boyle PA Buchman AS Wilson RS Leurgans SE Bennett DA. Physical frailty is associated with incident mild cognitive impairment in community-based older persons. J Am Geriatr Soc. (2010) 58:ds248–255. 10.1111/j.1532-5415.2009.02671.x 20070417 PMC3150526

[40] Wolinsky FD Bentler SE Hockenberry J Jones MP Obrizan M Weigel PAM. Long-term declines in ADLs, IADLs, and mobility among older Medicare beneficiaries. BMC Geriatr. (2011) 11:1–12. 10.1186/1471-2318-11-43 21846400 PMC3167753

[41] Royall DR Lauterbach EC Kaufer D Malloy P Coburn KL Black KJ. Thecognitive correlates of functional status: a review from the Committee on Research of the American Neuropsychiatric Association. J Neuropsychiatry Clin Neurosci. (2007) 19:249–65. 10.1176/jnp.2007.19.3.249 17827410

[42] Hughes AJ Beier M Hartoonian N Turner AP Amtmann D Ehde DM. Self-efficacy as a longitudinal predictor of perceived cognitive impairment in individuals with multiple sclerosis. Arch Phys Med Rehabil. (2015) 96:913–9. 10.1016/j.apmr.2015.01.008 25597915 PMC4410065

[43] World Health Organization the World Bank. World Report on Disability. (2011). Available online at: https://www.who.int/disabilities/world_report/2011/report.pdf (accessed July 29, 2021).

[44] National Alliance on Mental Illness (NAMI). Divert to What? Community Services That Enhance Diversion. (2020). Available online at: https://www.nami.org/Support-Education/ Publications-Reports/Public-Policy-Reports/Divert-to-What-Community-Services-that-Enhance-Diversion/DiverttoWhat.pdf (accessed July 29, 2021).

[45] Koslowski N Klein K Arnold K Koesters M Schuetzwohl M Salize HJ. Effectiveness of interventions for adults with mild to moderate intellectual disabilities and mental health problems: Systematic review and meta-analysis. Br J Psychiatry. (2016) 209:469–74. 10.1192/bjp.bp.114.162313 27198481

[46] Gustafsson C Öjehagen A Hansson L Sandlund M Nyström M Glad J. Effects of psychosocial interventions for people with intellectual disabilities and mental health problems: A survey of systematic reviews. Res Soc Work Pract. (2009) 19:281–90. 10.1177/1049731508329403

[47] Black BS Johnston D Morrison A Rabins PV Lyketsos CG Samus QM. Quality of life of community-residing persons with dementia based on self-rated and caregiver-rated measures. Qual Life Res. (2012) 21:1379–89. 10.1007/s11136-011-0044-z 22038392 PMC3296880

[48] Noyes K Bajorska A Wasserman EB Weinstock-Guttman B Mukamel D. Transitions between SNF and home-based care in patients with multiple sclerosis. NeuroRehabilitation. (2014) 34:531–40. 10.3233/NRE-141056 24463235

[49] Arling G Kane RL Valerie C Teresa L. Targeting residents for transitions from nursing home to community. Health Services Res. (2010) 45:691–711. 10.1111/j.1475-6773.2010.01105.x 20403058 PMC2875755

[50] Elias MF Dore GA Davey A Robbins MA Elias PK. From blood pressure to physical disability: the role of cognition. Hypertension. (2010) 55:1360–5. 10.1161/HYPERTENSIONAHA.110.149823 20404216

[51] Wehmeyer ML Abery BH. Self-determination and choice. Intellect Dev Disabil. (2013) 51:399–411. 10.1352/1934-9556-51.5.399 24303826

[52] Houseworth J Stancliffe RJ Tichá R. Examining the national core indicators' potential to monitor rights of people with intellectual and developmental disabilities according to the CRPD. J Policy Pract Intellect Disabil. (2019) 16:342–51. 10.1111/jppi.12315

[53] Shogren KA Ward MJ. Promoting and enhancing self-determination to improve the post-school outcomes of people with disabilities. J Vocat Rehabil. (2018) 48:187–96. 10.3233/JVR-180935

[54] Wehmeyer ML Palmer SB. Adult outcomes for students with cognitive disabilities three-years after high school: the impact of self-determination. Educ Train Dev Disabil. (2003) 2003:131–44.

[55] Wehmeyer ML Shogren KA. Self-determination and choice. In: Handbook of Evidence-Based Practices in Intellectual and Developmental Disabilities. (2016). p. 561–584.

